# Global overview of dietary outcomes and dietary intake assessment methods in maritime settings: a systematic review

**DOI:** 10.1186/s12889-021-11593-z

**Published:** 2021-08-21

**Authors:** Fereshteh Baygi, Fatemeh Mohammadi-Nasrabadi, Birgit-Christiane Zyriax, Olaf Chresten Jensen, Despena Andrioti Bygvraa, Marcus Oldenburg, Jesper Bo Nielsen

**Affiliations:** 1 Research Unit of General Practice, Department of Public Health, University of Southern Denmark, Odense, Denmark; 2 Food and Nutrition Policy and Planning Research Department, National Nutrition and Food Technology Research Institute (NNFTRI), Shahid Beheshti University of Medical Sciences, Tehran, Iran; 3 Midwifery Science – Health Care Research and Prevention, IVDP, University Medical Center Hamburg-Eppendorf, Hamburg, Germany; 4 Centre of Maritime Health and Society, Department of Public Health, University of Southern Denmark, Esbjerg, Denmark; 5 School of Community Medicine and Public Health, Department of Medicine, University of Gothenburg, Gothenburg, Sweden; 6 Institute for Occupational and Maritime Medicine (ZfAM), University Medical Center Hamburg-Eppendorf, Hamburg, Germany

**Keywords:** Systematic review, Food, Nutrition, Meal, Maritime settings

## Abstract

**Background:**

Seafaring is a risky occupation with high prevalence of risk factors for non-communicable diseases. Food intake and eating habits are important cornerstones regarding health and health promotion. The aim of this study was to provide an overview of dietary intake and dietary intake assessment methods of seafarers and suggestions for applicable assessment tools.

**Methods:**

We systematically searched PubMed and NLM Gateway (for MEDLINE), Web of Science, and SCOPUS up to February 2020 using standard keywords including nutrition OR diet OR meal AND maritime settings. Two independent reviewers extracted the data. The quality of included studies was assessed using the Joanna Briggs Institute Critical Appraisal checklist.

**Results:**

From 4449 studies initially identified, 26 articles were included in the final review. Qualitative data (e.g. on unhealthy eating) had been gathered using in-depth individual or group interviews, participant observations, and phone-based chats. Composition of menu analysis, 24 h dietary recall, food diaries/ diet records, dietary habits questionnaire, food stores and food waste of the ship were used to assess the quantitative outcomes (e.g. dietary intakes). Access to meat, processed meat and egg, frozen and canned food items, sugary drinks, alcohol, greasy and salty food was high. In contrast, consumption of fruit, vegetables, dairy products, and cereals was lower than recommended.

**Conclusions:**

Eating habits and dietary intakes in maritime settings are unhealthy. Subjective dietary assessment methods combining menu analysis with new technologies (e.g. mobile-based) might be an applicable method in this hard to reach setting which is the vessels.

**Supplementary Information:**

The online version contains supplementary material available at 10.1186/s12889-021-11593-z.

## Background

Living and working conditions on board affect the seafarers’ health [1, 2]. Previous studies have shown that seafaring is a risky occupation mostly characterized by a high prevalence of non-communicable diseases risk factors (NCDRs) [3–5]. Food intake and eating habits are important cornerstones with regard to seafarers’ health and health promotion. However, several studies indicate that overall food supply on board does not meet nutritional recommendations: Traditional food offer is often characterized by low quality and variety, predominately meat-oriented, while less vegetables, fruit and fish are served [6–10]. In addition, stressful conditions such as long working hours, less sleep, homesickness and irregular mealtimes influence appetite, emotional eating and promote the poor food choice [11–13]. This risk seems to grow with increasing duration of seafarer’s employment at sea. Consequently, overeating and preferences for energy-dense, low-fiber, high-starch, sugary, fatty and salty food are widespread problems in the Maritime settings [6–10].

In order to promote more balanced diets and reduce NCDRs, monitoring the daily nutritional intake on board is the first step and one of the feasible preventive methods given the maritime setting [1, 3, 4, 14–16]. To date, various tools (e.g. menu analysis, plate composition and the 24 h-dietary recall method) have been used [6–10, 15]. It is of high interest to apply a valid and reliable standard survey instrument, which is brief, easy to handle, cost-effective and allows the assessment and comparison of dietary intake in multi-ethnic crews. Thus, the main aim of the current review is to provide an overview of dietary outcomes and assessment methods in maritime settings. In this context, the current study is an inevitable prerequisite for future studies within Maritime Health and Safety Monitoring Programs.

This systematic review addresses the following research questions: 1. How are dietary intakes of seafarers in previous studies described? 2. Is it possible to identify indicators that best determine seafarer’s nutritional status? 3. Which tools have previously been used to investigate dietary intake in the maritime settings? And 4. Which instruments are the best suited to assess seafarer’s eating habits considering practical application as well as strengths and limitations of each method? Findings from this study will indirectly contribute to the sustainable development goals such as a valid documentation of seafarer’s nutrition status and initiation of relevant job training programs on the topic of healthy eating including training for the cooks. In the long-term, implementation of good working conditions and improvement of seafarer’s overall health will have a direct effect on their productivity and increase economic growth both for themselves and the industry.

## Methods

### Identification of relevant studies

**Fig. 1:**
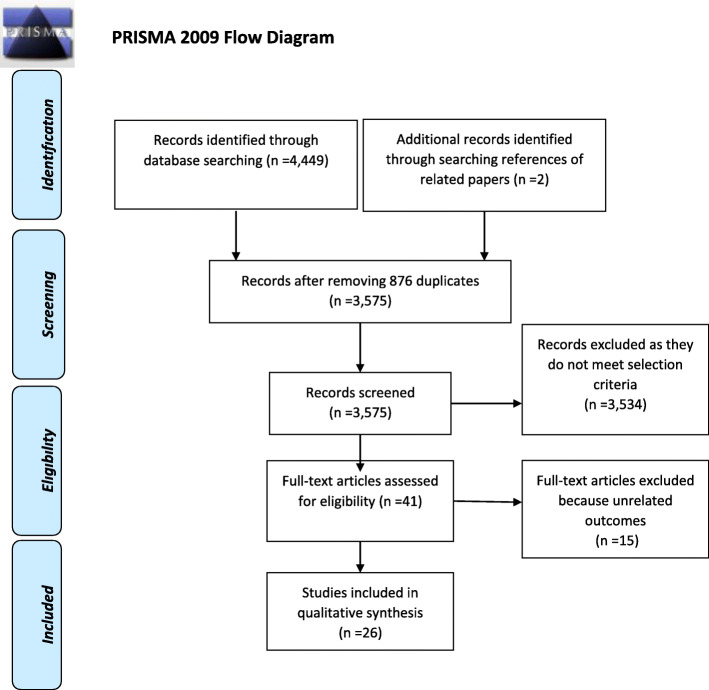
PRISMA 2009 flow diagram. *From:* Moher D, Liberati A, Tetzlaff J, Altman DG, The PRISMA Group (2009). *P*referred *R*eporting *I*tems for *S*ystematic Reviews and *M*eta-*A*nalyses: The PRISMA Statement. PLoS Med 6(7): e1000097. doi:10.1371/journal.pmed1000097

The current systematic review followed the PRISMA-P guideline (Fig. 1) [17]. All documents are based on the details of the study protocol. The registration number of the study in the International Prospective Register of Systematic Reviews (PROSPERO) is CRD42020173653.

The root of developing the search strategy is based on the two main components: “dietary intake” and “seafarers”. There was no limitation for language and publication date. All studies published until the end of February 2020 were included in the study. For documents on other languages than English, necessary arrangements were taken for their translation. To reach the optimal sensitivity of searching for documents, we simultaneously searched the most comprehensive database including PubMed and NLM Gateway (for MEDLINE), Web of Science (ISI/WOS), and SCOPUS as the main international electronic data sources (Additional file 1). Moreover, reference lists of included studies or reviews were studied to identify more and older potentially eligible studies.

### Inclusion and exclusion criteria

This study presents a comprehensive overview of the works that have been published so far by focusing on dietary intake of seafarers including variety of food and on different tools which have been used to assess dietary intake. Studies with outcomes such as different food groups intake, vitamin and mineral intake, satisfaction with diet onboard and other outcomes complying with the objective of the study in the target population were included. Besides, studies with outcomes like body composition and biomedical indexes- as surrogates to measure the dietary intake- were included [18]. All relevant results were extracted from cohort, cross-sectional, retrospective, surveys, before- after and qualitative studies. There was no limitation for the target groups in terms of age or gender of published studies. Book chapters and available conference proceedings were also considered for more access to relevant data. Duplicates and non-peer reviewed publications were excluded. Moreover, six old publications that were not accessible were excluded from the study [19–24].

### Quality assessment and data extraction

Two independent reviewers conducted the systematic literature review process, quality assessment, and the data extraction of eligible papers. Any discrepancies were resolved by a third reviewer.

The quality of included studies was assessed using the Joanna Briggs Institute (JBI) Critical Appraisal checklists for prevalence studies, quasi- experimental (non-randomized experimental) studies, and qualitative evidence. The quality of each study was assessed and rated high (H), medium (M), or low (L) based on the number of Yes options selected from the checklists [25]. A score of 0–3 was considered as low, a score of 4–6 as medium, and a score of above 6 as a high-quality study.

The extracted data included author and year of publication, population characteristics (mean age/age range and subjects), and methodological characteristics (study design, sample size, sampling method, type of ship or shipping sector, tools for measurements and outcomes).

### Statistical analysis

Data synthesis was the main strategy. The heterogeneity of the included studies in terms of the participants, study methods, and outcome measurements hampered the possibility of a meta-analysis. Therefore, the results were presented as qualitative and quantitative syntheses according to the type of the study.

## Results

### Study selection process

A total of 4449 studies were identified by the initial search and 2 additional records identified through searching references of the related papers. After the removal of 876 duplicates and excluding 3534 articles which did not meet selection criteria, 41 studies remained. Of them, 15 articles were excluded after the full-text review, because the outcomes did not comply with the objectives of the current study (Fig. 1). Finally, 26 studies were eligible for inclusion in this systematic review.

### Study characteristics

Table placed in additional file 2 shows the characteristics of the included studies on diet in maritime settings and the mostly used tools to assess these. Of included studies -in the period of 1970 to 2019- the US researchers conducted the most studies [26–31]. Other countries according to the number of studies were as follow: UK [32–35], Germany [10, 36, 37], Denmark [8, 38], Iran [39, 40], India [41, 42], China [43], Croatia [44], Italy [45], Brazil [46], Philippines [47], Lithuania [48], and Poland [6]. However, it appears that some of these studies from Germany [10, 36, 37], Denmark [8, 38], Iran [39, 40] and the US [27, 29] are sub-projects conducted within the framework of a larger and more comprehensive program.

In terms of study design, the majority of the studies (12) were cross-sectional [10, 27–30, 32, 34, 36, 40, 44, 45, 48], whereas 3 of them were designed as qualitative [39, 43, 47], three studies as pre-and post/interventional [8, 38, 42], one reported retrospective design with existing data [46] and 7 studies did not report the study design [6, 26, 31, 33, 35, 37, 41]. At least two studies applied mixed methodology which probably was presented in separate articles [8, 38–40].

Sample size ranged from 12 participants in a qualitative study [47] to 2060 in a quantitative survey [45]. Sampling method was not mentioned in 13 articles [6, 8, 26, 30, 33–37, 41, 45, 46, 48]. However, most of the studies sampled all available subjects through recruiting/voluntarily [10, 27, 29, 32, 38, 42–44]. Three studies applied random sampling [28, 31] or cluster random sampling [40]; Whereas two studies used purposive sampling because of their qualitative nature [39, 47].

### Qualitative synthesis

#### Tools and dietary outcomes

**Fig. 2:**
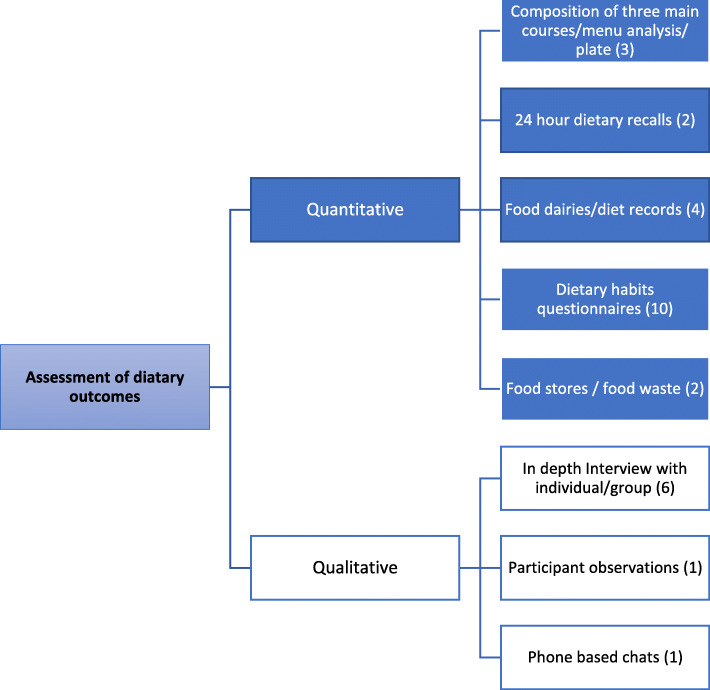
The most commonly used tools to assess dietary outcomes in maritime settings based on studies included in systematic review

Figure 2 summarizes the different tools for dietary assessment used in the studies included.

##### Qualitative tools and outcomes

Qualitative data in the reviewed articles were collected by using the following tools: in depth individual/group interviews [8, 10, 36, 37, 39, 47], participant observations [8], and phone-based chats [43].

Qualitative dietary and non-dietary outcomes of included studies were unhealthy eating and inappropriate dietary plan, living experiences in dealing with maritime health issues, self-perceived changes and possible barriers for changes.

##### Quantitative tools and outcomes

The following quantitative tools were used for dietary assessment: composition of main courses/menu analysis [6, 10, 42], 24 h dietary recalls [10, 26], food diaries/ diet records (1, 4, 7 or 8 days) [27, 33–35], dietary habits questionnaire (even one or two questions) [8, 28, 30–32, 38, 40, 44, 45, 48], and food stores and food waste of the ship [35, 41]. Additionally, health parameters related to nutrition such as anthropometric measurements [26, 27], bioelectrical impedance analysis (BIA) [42], blood samples and the 24-h urine collections [27, 29] were used.

Quantitative dietary outcomes measured included quality and quantity of diet in the three following categories:

##### Dietary intakes

Access to/ consumption of different food groups, the comparison of energy, macro- and micronutrients intake with dietary guidelines.

Our findings revealed that access to meat, processed meat and egg, frozen and canned food items, sugary drinks, alcohol, greasy and salty food is high in maritime settings. In contrasts, consumption of fruit, vegetables, dairy products, and cereals is lower than recommendations according to respective guidelines [6, 10]. Also, higher amount of energy derived from fats, especially saturated ones, and lower from carbohydrates was reported [6] and dietary intakes did not meet reference values of micro-nutrients and fiber [30, 33].

##### Eating habits

Nutritional habits included information on the frequency of balanced healthy eating, tendency to overeat, eating pattern, food choices, drinking beverages.

Most of the studied sample reported overeating, inappropriate dietary habits and westernized dietary pattern [32, 37, 44, 47], and made no attempt to eat healthy [32].

##### Body composition and biomedical indexes

Body fat percent and biomedical indicators included total cholesterol [9], high density lipoprotein (HDL), Sodium (Na), Potassium (K), Magnesium (Mg), Zinc (Zn), and Copper (Cu) levels. Although mean intakes exceeded Recommended Dietary Allowances (RDAs), serum levels of Mg, Zn, and Cu among 34, 44, and 37% of Navy trainees were below recommendations, respectively [29]. Urinary Sodium excretion was high. However, Potassium and selected vitamin levels approximated the military RDA [27].

The satisfaction with the meals served on board, food preferences and knowledge about healthy diet/foods are additional quantitative outcomes measured in some reviewed studies [10, 32, 36, 37].

### Quality of the studies

The results of quality assessment of the included studies are presented in Table 1. Out of the 26 included studies, 21 (80.8%) were rated as high quality. The remaining 5 studies were rated as moderate quality mostly due to not presenting the required data or weak design (e.g. not using random allocation, not reporting the validity and reliability of the study instrument, and high non-responding rates). Only one study met all criteria required for the rating of quality.

**Table 1:** Strengths, limitations and feasibility of dietary assessment methods at sea (Adapted from [18] additionally with authors’ opinion

Methods	Limitations	Strengths	Feasibility at sea
**24-h dietary recalls**	Recall bias, trained interviewers required, Interviewer bias, multiple days required to assess usual intake	Provides detailed intake data; relatively small respondent burden (literacy not required).	Would be applicable by using interactive computer-based technology.
**Dietary records**	Respondents should be trained before the study, respondents should have high level of motivation, possible under-reporting, expensive and time-consuming; multiple days required to assess usual intake	Provides detailed intake data; no interviewer required; no recall bias	Would be applicable by using interactive computer-based technology.
**Dietary history**	Trained interviewers required, time consuming, high costs	Assess usual dietary intake over a long period of time, self-administrative	Not applicable because of complicated measurements and possible related errors. Also, it would be problematic for application among multi ethnicity groups.
**Food frequency questionnaire**	Specific to study groups and research aims; uses a closed-ended questionnaire; low accuracy (recall bias); requires accurate evaluation of developed questionnaires	Assesses usual dietary intake simply, cost-effective and time saving, suitable for epidemiological studies. Requires a certain degree of literacy.	Might be applicable by using interactive computer-based technology. But factors including culture, ethnicity and individuals’ preferences that can influence diet should be considered precisely in development of the questionnaire.
**Food consumption record**	Trained staff required, not suitable among those who eat in group	Ease of application among those with low literacy.	Not applicable among those who eat in group.

### Discussion

Our review included 26 studies on dietary intakes which were based on either qualitative or quantitative research methodologies. Qualitative dietary outcomes were unhealthy eating and inappropriate dietary plan. Assessed quantitative dietary outcomes were classified into the following categories: Dietary intake, eating habits, body composition and biomedical indexes.

Overall, the reviewed evidence tends to show that dietary intake and eating habits in maritime settings in many cases are unhealthy. Also, future health intervention programs regarding healthy eating and proper food choice has been recommended.

Workplace is a main venue influencing dietary habits even in land-based occupations. Thus, studies conducted on nutritional status and eating habits of shift workers revealed that working in such rotating time schedule decreased fruit and vegetable intakes and increased intakes of high fats and fast foods [49, 50]. In the other words, these individuals -including seafarers- are more vulnerable to dietary risk factors of NCDs potentially due to difficulties in finding healthy foods when needed. Also, comparing the nutrient intake with the recommended values in a study conducted on bus drivers showed that both day and night shift workers had poor diet [50]. Since poor diet is a leading risk factor for obesity and metabolic-syndrome, worksite health promotion programs are highly recommended both in land-based and maritime jobs.

Current systematic review revealed that different qualitative and quantitative tools have been used to describe seafarers’ diets. Among such tools, dietary habits questionnaire was the mostly used tool to assess dietary outcomes in maritime settings. However, validation studies of this tool were rarely performed which can influence the accuracy of the results. No reviewed studies have used food frequency questionnaire (FFQ), which requires recall over a long period. Strengths, limitations and feasibility of different dietary assessment tools at sea adapted from [18] and additionally with authors’ opinion are summarized in Table 1.

Selection of an appropriate method for dietary assessment depends on the purposes of the study, which may be to measure the food consumption, nutrient intake or eating habits [51]. On the other hand, the methods should be tested to assure if they are accurate and reliable for the study population [52]. The authors of the current systematic review believe that within this maritime occupational setting, using a few questions to assess dietary habits of the participants may not capture the full picture of nutritional status or dietary intakes of the participants. Also, we think the main reasons for using the mentioned questionnaires in this setting is that it is the simplest method for such hard to reach population, and because filling out a questionnaire can be done by the respondents independently and without any training. However, there is a need for studies on the quality and validity of such methods. Further, in maritime settings, multi-cultural crew members with different anthropometric indicators, biometric parameters (e.g. blood values) and nutritional habits live and work together. Thus, for more accuracy and reliability of the results in the future, we suggest dietary assessment methods (e.g. valid dietary habits questionnaire) combining with anthropometric measurements which are feasible in this moving workplace. Anthropometric indicators will provide reliable information on weight changes and can assist in assessing the nutritional status of the studied population [53].

According to our findings, the first studies have been conducted in this setting to demonstrate vitamin deficiencies. In other words, since the 70s, studies conducted in maritime settings have focused on more general aspects of diet like macro and micronutrients intake, especially vitamin C of the sailors [19]. Such studies were initially introduced by the United Kingdom [33, 35] and then by the United States [27, 29, 30]. Also, the first studies of nutritional status of land-based workers focused on nutrient deficiencies. For example, a study conducted on industrial workers (in 1954) provided evidence about suboptimal nutrition with respect to one or more nutrients [54].

A recently published study on the history of modern nutrition revealed that nutrition is quite young science so that, in the first half of the twentieth century the focus of the studies was on single nutrient deficiency diseases [55]. This present literature study also showed that over the past three decades the role of nutrition in reducing the risk for non-communicable diseases has been more highlighted [55]. Global shifts in consumption patterns called nutrition transition (e.g. increases in food consumption and a higher tendency for consuming fast food) appears to be the best explanation for such focus of the nutrition research in recent decades [56]. The mentioned shifts in types of the nutritional studies are obvious on the evaluation of nutritional status of general population and land-based occupations as well [57, 58]. In addition, a variety of the tools have been used to collect nutritional data on land-based jobs [59]. But in maritime settings, most of the included studies had descriptive cross-sectional design, while a well-designed cohort or interventional studies is required [58].

There have been very few studies of assessing the relationship between diet and non-communicable diseases at sea [3, 39, 40]. In such studies, only one or two tools has been used to evaluate the dietary intake of the participants [39]. Also, most of them failed to use the advanced nutritional analysis which is recently more common in studies within general populations or land-based job settings [57–59]. This might be because employees of the maritime setting are hard to reach. So, BIA, blood samples and 24-h urine collections as additional health parameters related to nutrition can be hardly used in the maritime setting. Furthermore, due to logistic position of the ships, the presence of researchers on board at sea for data collection with different tools is staff intensive and costly. Therefore, prospective studies and applicable advanced analysis (e.g. healthy eating indicators) are required to examine the possible relationship of diet to health-related problems in this occupation. Present development within the area of on-site, easy-to-use, sampling and analysis of biomedical parameters may potentially pave the way for future studies on dietary habits and health outcomes in the maritime setting. For instance, home use tests which are cost effective and quick might allow individuals in maritime setting to test biomedical parameters (e.g. cholesterol, blood glucose levels) independently. Consequently, they will be able to more frequently monitor at-sea health conditions. But further studies in order to examine the possibility, accuracy of the results and challenges of using such home-based kits in this occupation is highly recommended.

### Limitations and strengths of the study

To the best of our knowledge, this is the first review study to provide an overview of dietary outcomes and dietary intake assessment methods used in maritime settings. The results of this study can be used to further exploration of the healthy eating behavior of seafarers, so as to provide suggestions for promoting healthy eating habits. However, lack of access to some studies, especially old publications, moderate quality of some studies, high frequency of duplicate data, and heterogeneity of studies can be considered as limitations of the study which may influence the study results. Besides, some of the included studies have no restrictions on the age and gender of the target population; this might cause the results of the analysis to fail to meet expectations. Also, due to the particularity of seafarers’ occupations, there are relatively few researches on seafarers, and conducting questionnaires may lead to insufficient validity of the result. Also, mostly same type of the research has been done at home and on- board. So, insufficient samples of the literature, and the high frequency of repeated data may affect the research results.

## Conclusions

The results obtained from the present study revealed that dietary intake and eating habits in maritime settings are unhealthy. Among different tools, the dietary habits questionnaire is commonly used to assess dietary outcomes in this occupation, however, under the professional background of seafarers, the surveys which uses several questions to evaluate the dietary habits of the participants, may not be able to fully understand the nutritional status or dietary intake of the participants and using combination of methods is recommended.

Based on our findings, to provide assistance in the collection of dietary data in maritime settings, subjective dietary assessment methods (e.g. dietary records or multiple recalls) combining menu analysis with new technologies (e.g. mobile applications) might be an applicable method in this hard to reach occupation on board. Dietary assessment methods that utilize technology offer many advantages for research and are often preferable to consumers over more traditional methods [60]. The image-assisted methods can improve the accuracy of conventional dietary assessment methods by adding eating occasion detail via pictures captured by an individual (dynamic images) [61]. Therefore, the food items consumed are obtained individually from dietary records or recalls, and the components of food prepared in the ship’s kitchen are completed by analyzing the food menu. So, data are collected by self-report approach, actual intakes on specific days will be collected, and the burden of memory may be less compared to the food frequency questionnaire; however, high level of motivation is required and tendency to under-reporting might be observed. Dependent on the purpose of the dietary assessment, dietary habit questionnaires or qualitative tools can be used, too. However, validity and reliability of these instruments should be considered to facilitate and improve the quality and accuracy of nutrition data and indicators in maritime settings.

Advanced statistical methods e.g. factor analysis for dietary patterns or more complex indicators e.g. healthy eating index (HEI) or dietary diversity (DD) will be helpful to analyze the association between dietary intakes and non-communicable diseases in maritime settings. Likewise, the continuous development of test kits for biochemical markers in blood, urine, or saliva should be followed carefully as such measurements could strengthen conclusions on associations between dietary habits and health outcomes of seafarers.

## Supplementary Information

**Additional file 1.** Details of search strategy. This file provides a detailed description of the search strategy used for finding studies.

**Additional file 2.** Characteristic of included studies. This file provides the characteristics of the included studies in a table form, including first author, year of publication, country, study design, study subject, sample size, sampling method, mean age/age range, setting, tools for measurements, outcome, result and quality rate.

## Data Availability

Since no dataset were generated during the current study, data sharing is not applicable.

## Abbreviations

NCDRs: Non-communicable diseases risk factors
PRISMA: Preferred reporting items for systematic reviews
PROSPERO: Prospective register of systematic reviews
WOS: Web of science
JBI: Joanna Briggs institute
BIA: Bioelectrical impedance analysis
HDL: High density lipoprotein
Na: Sodium
K: Potassium
Mg: Magnesium
Zn: Zinc
Cu: Copper
RDAs: Recommended dietary allowances
FFQ: Food frequency questionnaire
HEI: Healthy eating index
DD: Dietary diversity
