# Supplementary material for: Global overview of dietary outcomes and dietary intake assessment methods in maritime settings: a systematic review
Source: BMC Public Health. 2021 Aug 21;21:1579. doi: 10.1186/s12889-021-11593-z (PMC8379789; doi:10.1186/s12889-021-11593-z)
Supplement: Supplementary file 1 — Additional file 1. Details of search strategy. This file provides a detailed description of the search strategy used for finding studies. [file 12889_2021_11593_MOESM1_ESM.docx]

**Additional file 1.** Details of search strategy

| **PubMed** |
| --- |
| (("Nutrition"[Title/Abstract] OR "Food"[Title/Abstract] OR "Nourishment"[Title/Abstract] OR "Meal"[Title/Abstract] OR "Dish"[Title/Abstract] OR "Intake"[Title/Abstract] OR "Diet*"[Title/Abstract]) AND ("Seafarer"[Title/Abstract] OR "Sailor"[Title/Abstract] OR "Navigator"[Title/Abstract] OR "Seagoing"[Title/Abstract] OR "Mariner"[Title/Abstract] OR "Maritime"[Title/Abstract] OR "Navy"[Title/Abstract] OR "Shipper"[Title/Abstract] OR "Seaman"[Title/Abstract] OR "Submarine"[Title/Abstract])) NOT "Navy bean"[Title/Abstract]) |
| **Scopus** |
| (TITLE-ABS-KEY ( "Nutrition" OR "Food" OR "Nourishment" OR "Meal" OR "Dish" OR "Intake" OR "Diet*" ) AND TITLE-ABS-KEY ( "Seafarer" OR "Sailor" OR "Navigator" OR "Seagoing" OR "Mariner" OR "Maritime" OR "Navy" OR "Shipper" OR "Seaman" OR "Submarine" ) AND NOT TITLE-ABS-KEY ( "Navy bean" ) ) |
| **ISI/WOS** |
| ("Nutrition" OR "Food" OR "Nourishment" OR "Meal" OR "Dish" OR "Intake" OR "Diet*") AND TOPIC: ("Seafarer" OR "Sailor" OR "Navigator" OR "Seagoing" OR "Mariner" OR "Maritime" OR "Navy" OR "Shipper" OR "Seaman" OR "Submarine") NOT TOPIC: ("Navy bean")  Timespan= All years. AND  Indexes: SCI-EXPANDED, SSCI, A&HCI, CPCI-S, CPCI-SSH, BKCI-S, BKCI-SSH, ESCI, CCR-EXPANDED, IC= All years. |
