# Supplementary material for: Global overview of dietary outcomes and dietary intake assessment methods in maritime settings: a systematic review
Source: BMC Public Health. 2021 Aug 21;21:1579. doi: 10.1186/s12889-021-11593-z (PMC8379789; doi:10.1186/s12889-021-11593-z)
Supplement: Supplementary file 2 — Additional file 2. Characteristic of included studies. This file provides the characteristics of the included studies in a table form, including first author, year of publication, country, study design, study subject, sample size, sampling method, mean age/age range, setting, tools for measurements, outcome, result and quality rate. [file 12889_2021_11593_MOESM2_ESM.docx]

**Additional file 2.** Characteristics of the included studies on dietary outcomes and assessment methods used in maritime settings.

| **Author, Year, of publication, Country** | **Study design** | **Study subject** | **Sample size** | **Sampling method** | **Mean age/ Age range** | **Setting (type of ship, shipping sector)** | **Tools for measurements** | **Outcome** | **Results &significancy** | | **Quality rate†** |
| --- | --- | --- | --- | --- | --- | --- | --- | --- | --- | --- | --- |
| von Katzler et al. ^(36)^, 2019, Germany | CS | Seafarers  -Non-EU  -EU | 81 | NP | 20-64 | Merchant ships | -Interview on nutritional behavior  -Blood samples | Attitudes to weight, shape and nutrition  and disinhibition of control as a characteristic of eating behavior, BMI | Non-Europeans often claimed to eat significantly larger amounts of food aboard, while most European sailors stated that they ate less or about the same during their shipboard  stay | | H |
| Zyriax et al. ^(10)^, 2018, Germany | CS | Seafarers  -Non-EU  -EU | **T:** 81  -**Non-EU:** 48  **-EU:** 33 | Recruiting | 20-64 | Merchant ships | -Composition of three main courses on 7 consecutive days;  -3 non-consecutive 24-h dietary recalls | -Quantity of diet  -Quality of diet | **-**Supply higher than recommendation:  Meat, processed meat and egg  -Lower than recommendation:  Fruit, vegetables, dairy products, cereals  -Both groups did not reach reference values of macro- and micronutrients, and fiber intake. | | H |
| Westenhoefer et al. ^(37)^, 2018, Germany | CS | Seafarers  -Non-EU  -EU | 81 | NP | 37.85 | Merchant ships | -Questionnaire (TFEQ)  -Interview | -Tendency to overeat  -Views on the food & nutrition on board | -More tendency to overeat (Non-EU)  -A highly tempting westernized food  environment | | H |
| Baygi et al. ^(39)^, 2018, Iran | Qualitative | Seafarers | 17 | Purposive | 35.8 | Oil tanker | Interview | -Unhealthy eating  -Inappropriate dietary plan | -Low access to fruit and vegetables  -High consumption of frozen and canned food items  -Greasy and salty food | | H |
| Baygi et al. ^(40)^, 2017, Iran | CS | Seafarers | 231 | Cluster Random Sampling | 35.9 | Oil tanker | Questionnaire (HPLPII) | Nutrition behavior | Lower nutrition score among engine department (S) | | H |
| Zhang and Zhao ^(43)^, 2017, China | Qualitative | Seafarers | NP | Voluntary | NP | Shanghai Marine  Shipping Association | Semi-structured interviews with a range of key stakeholders followed by phone-based chats | Experience in dealing with maritime health issues | Most Chinese ships only spend one third to one half of the provision subsidies on the crew's food and all the savings are distributed to improve the crew's income. It may impair the standard of food. | | M |
| Slišković ^(44)^, 2017, Croatia | CS (On-line survey) | Seafarers | 530 | Voluntary | 37.7±9.5 | Associates from related organizations | Questionnaire | Self-rated frequency of healthy balanced diet | Unhealthier diet at sea than at home. | | H |
| Gunner et al.  ^(34)^, 2017, UK | CS | Navy | **SUBM1:** 36  **SUBM2:** 60 | NP | NP | Submarines | 4-day food diary | -Total energy & macronutrient intake  -Saturated fat intake | Total energy intake was lower than recommendations for general population. | | H |
| KnapiK et al.  ^(28)^, 2016, US | CS | Navy | 1,683 | RS | 18≤X ≤40 | Navy& Marine Crops | Questionnaire | Dietary habit on using caffeinated beverages | **•Commonly used caffeinated beverages:**  -Coffee: 65%  -Cola:54%  -Tea:40%  -Energy drinks:28%  **•Mean caffeine uses**  -226±5mg/d | | H |
| Mahdi et al.  ^(45)^, 2016, Italy | CS | Seafarers | 2,060 | NP | NP | All type of the ships | Questionnaire | Dietary habits | -Regular use of fermented carbohydrate (dairy products and candies): 55.7% (1,147)  -Alcohol use: 11.5% (236) | | H |
| Hjarnoe and Leppin^(8)^, 2014, DK | Intervention  study | Cooks  Seafarers | 49  630 | All | NP | 2 Danish shipping companies | - Group interviews and participant observations  - One question | - Self-perceived changes  - Frequency of eating healthy  - Possible challenges | Greater number claimed to eat healthily on more days of the week at Time 2 (diet of green, high-fiber and low-fat products) | | H |
| Hjarnoe and Leppin  ^(38)^, 2013, DK | Pre-post design | Seafarers | **B:** 343  **F:** 209 | NP | **B:** 42  **F:** 44 | Two Danish shipping companies | Questionnaire Two questions: | Dietary habit  Frequency of overeating and intake of sugared products | Decrease in daily sugar intake | | H |
| Darnell et al.  ^(26)^, 2013, US | NP | Active duty SEALs | 215 | NP | 29.7± 6.8 | NP | Anthropometric measurements on the same day as the 24-hour recall | Total energy (kcal), carbohydrate, protein, fat, saturated fat (g%), fiber (g), cholesterol (mg), K (mg), Ca (mg), vitamin D (IU) | Low intake of CHO and other key nutrients except for Ca, high in fat, saturated fat, and cholesterol | | H |
| Almeida ^(46)^, 2012, Brazil | Retrospective, existing data | Correspondence from officers and  of the Navy ministers | NP | NP | NP | NP | Letters and reports by Navy Ministers | Information about the health and food of Brazilian Navy at the beginning of the 20th century | The main products of the diet to be on board were proteins, like meat, or carbohydrates, like bread, cookies, rice, potatoes and sugar. Crews faced the shortage of fresh food, coal and drinking water | | M |
| Gregorio ^(47)^, 2012,  Philippines | Qualitative | Seafarers | 12 | Purposive | 18≤X | Industrial clinic | In depth interview | The lived experiences about seafarers’ health conditions | Unlimited amount of food servings available, the lack of control over food choices, and negative attitude of the chief cook as increasing vulnerability to NCDs | | H |
| Singh, et al. ^(42)^, 2011, India | Before and after | Crew of:  -Warships  -Submariners | 35  20 | Voluntary | 24.8± 5.2  26.5± 3.8 | Eastern Naval Command | The duplicate plate samples of breakfast, lunch, and dinner for 14 days, Bioelectrical Impedance Analysis (BIA) | Energy expenditure, nutrient intake, level of nutrients in body, urinary excretion, and  changes in body composition | No sign and symptoms of any nutritional deficiency were observed either initially or after three months of nutritional monitoring. | | H |
| Salyga and Zabrocki ^(48)^, 2005, Lithuania | CS (Pilot study) | Seafarers | 997  993 | NP | NP | Medical Examination Center | Questionnaire containing of 44 questions | Nourishment on ship | Latvian seafarers took more bread, boiled and roast potatoes, porridge or flakes, cheese, meat, chicken, sound fruits and vegetables, eggs, coffee, juice, sugar and salt. | | H |
| Babicz-Zielińska ^(6)^, 1998, Poland | NP | -Seamen  -Fishermen | 55  36 | NP | NP | -Sea-going vessels  -Deep-sea fishing ships | 1-Average food intake  2-Daily calorie intake  3-Quality assessment of the 112-week menus | Food intake for seamen  Daily calorie intake | 1- The excessive consumption of eggs, meats and sausages, butter and sour milk, processed vegetables and potatoes;  2- High calorie intake, higher energy derived from fats and lower from carbohydrates than recommended;  3- Insufficient fresh vegetables & fruits, monotony in choice of products and excessive fried and baked meats | | M |
| Woodruff and Conway  ^(31)^, 1994, US | NP | **•**Navy Personnel  **•**Shore-based service members | 747 | RS | 29.1 | Navy | Questionnaire  (HPTF) | Eating habits | -More than 50% of samples had skipped breakfast  -Overeating in more than 10% of samples  -Less frequent consumption of high fat meat & high fat dairy products compared to 1988  -More frequent consumption of fish & vegetables compared to 1988 | | H |
| Singh et al. ^(29)^, 1989, US | CS | US Navy SEAL trainees | 270 | Recruiting | 22.5 ± 0.2 | Three  training classes at the SEAL  School | One-day diet records, blood samples, 24-h urine collections | Mean dietary intake and mean plasma concentration of magnesium, zinc, and copper | Although mean intakes exceeded RDAs or range, intakes of 34%, 44%, and 37% of the trainees were below the recommendations for Mg, Zn, and Cu, respectively. | | H |
| DeBolt et al. ^(27)^, 1988, US | CS | US SEAL trainees | 267 | Sampling of  states and territories in the US | 22. 1 ± 0.3 | Three separate training classes | The Navy circumference method, Diet records, blood samples, 24-h urine collections | Percent body fat, energy, micro, and macronutrients, Serum cholesterol, HDL, Na, and K concentrations | Fat, cholesterol, and Na intakes were higher than the dietary goals. Urinary Na excretion was high and correlated with Na intake. Potassium and selected vitamin intakes approximated the Military RDA. | | H |
| Trent and Conway ^(30)^, 1988, US | CS | Navy | 1,013 | NP | 26. 2 ±6.3 | Navy ships | A self-report  survey of lifestyle and dietary habits | Eating Patterns (breakfast, lunch, dinner, snacking,  overeating, and fasting), food, caffeine and alcohol | The participants tended to skip breakfast, ingest moderate amounts of caffeine, and favor a high-fat, low-fiber diet. | | H |
| Bull  ^(32)^, 1988, UK | CS (Survey) | Royal Navy | 196 | All listed personnel | NP | Navy serving | A questionnaire elicited of attitudes towards and habits of alcohol consumption, smoking, exercise and diet. | Attempting control of diet in four areas (fats, fiber, sugar, cholesterol) using foods such as eggs, confectionery, milk, cheese, margarine/butter, fried foods, whole meal/white bread, fruit, potatoes, vegetables | Alcohol consumption amongst members of the Royal Navy is higher than amongst civilians (72% moderate or heavy as against 51%), while 26% made no attempt to eat healthily rather than otherwise. | | H |
| Malhotra et al. ^(41)^, 1976, India | NP | Submariners | NP | NP | 23-31 | Submarine | -Actual Food intake with inventory method, energy expenditure, food wastage | **Mean daily** dietary intakes in balance to energy expenditure | **-Phase I*:**  - Energy:12.45 MJ  -Protein:103gr  -Fat: 90 gr Carbohydrate:450gr  -Vitamin A:1,334 µg  -B1:2.6 mg  -B2:3.8 mg  -B3:32 mg  -Vit C:181 mg  -Iron:52 mg  -Ca: 1.23 gr | **-Phase II****  -11.80 MJ  - 80 gr  - 90 gr  - 422 gr  - 1356 µg  -2.1 mg  - 2.9 mg  - 23 mg  - 147 mg  - 41 mg  - 0.98 gr | M |
| Eddy et al.  ^(33)^, 1971, UK | NP | Seafarers | 6 | NP | NP | Oil tanker | 8 days detailed dietary intake | Macro& Micronutrient intake | **•Macronutrient intake for the whole ship:**  -Protein: 12%  -Fat: 35%  -Carbohydrate: 40%  **•Nutrient intake for each person:**  **-**Vitamin C: 70 mg/d  -Thiamine intake deficiency | | M |
| Southgate and Shirling ^(35)^, 1970, UK | NP | Submariners | 13 | NP | NP | Submarine | -Food stores  -Food waste  -Individual Food record three days | -Food composition  -**Dietary intakes** | **•Net Intake:**  **🞄Six-day period:**  - Energy: 3,711 Kcal  -Protein:103 gr  -Fat:157 gr  -Carbohydrate:415gr  **•Mean daily dietary intake:**  **🞄Period 1:**  - Energy: 3,844 Kcal  -Protein:110 gr  -Fat: 203 gr  -Carbohydrate:372 gr | **🞄Four-day period:**  -3376Kcal  - 99 gr  - 153 gr  - 339 gr  **Period 2:**  -3015Kcal  - 107 gr  - 144 gr  - 277 gr | H |
| **CS:** Cross-sectional, **NP**: Not-provided, **T:** Total, **TFEQ:** Three-Factor Eating Questionnaire, **Non-EU**: Non-European, **EU**: European, **HPLPII:** Health Promotion Lifestyle Profile, **NA**: Not applicable, **S:** Significant, **mg:** milligram, **d:** day, **SUBM**: Submarine, **B:** baseline, **F:** follow up, **RS:** Random sampling, **MJ:** Mega Joules, **gr:** gram, **µg:** microgram, **Kcal:** Kilo calorie, **HPTF**: Health Promotion Tracking Form, **SEAL**: Navy Sea, Air, and Land, **RDAs**: the Recommended Dietary Allowances, **ESAI**: the Estimated Safe and Adequate Intake  **†** **Quality rating**: High (H), medium (M), low (L)  *Precooked, preserved and ready to eat rations were issued.  ******Rations served were cooked onboard. | | | | | | | | | | | |
